# Insights into ThB_40_: Stability, Electronic Structure, and Interaction

**DOI:** 10.3390/molecules29061222

**Published:** 2024-03-08

**Authors:** Yutian Li, Yingying Wang, Zhanrong Zhou, Yang Gao, Yiming Chen, Guoqing Zhang, Chao Ma

**Affiliations:** 1 Xi’an Research Institute of High Technology, Xi’an 710025, Chinagaoyang_nudt@126.com (Y.G.);; 2 Foundation Department, Engineering University of PAP, Xi’an 710086, China

**Keywords:** density functional theory calculations, boron–thorium interaction, electronic structure

## Abstract

The interaction between nonmetal and metal atoms has attracted great interest in the development of organometallic compounds and their promising applications. In this study, we explored the interaction between boron and thorium atoms, based on the stable B_40_Th coordination compound, by employing density functional theory calculations. We elucidated the stability and geometries of the B_40_Th coordination compound and revealed the electron transfer from the metal atom Th to B_40_, which is evidenced by the natural bond orbital calculations. This electron transfer is attributed to the electron-withdrawing character of the boron atom and results in clear electrostatic interaction. Additionally, bond critical analysis and bond order calculations show obvious covalent characters between the metal and nonmetal atoms. The IR spectrum was simulated to give detailed information to identify this targeted compound in future experiments. This study is expected to enhance the understanding of metal–nonmetal interactions and provides useful information for constructing new organometallic compounds based on actinium metal atoms.

## 1. Introduction

The interaction between metal and nonmetal atoms, particularly actinium metal atoms, is complicated due to the complex electronic structure of metal atoms [1,2,3]. Understanding these metal–nonmetal interactions is crucial for the development of organometallic compounds [4,5,6], which have a wide range of promising applications in energy, environment, and information fields. This has attracted much attention and prompted further research into metal–nonmetal interactions to prepare new organometallic structures.

In the last few decades, endohedral metallofullerenes have been regarded as the ideal models to study the interaction between encaged metal atoms and nonmetal atoms, including actinium metal atoms [7,8,9,10,11,12,13]. The discovery of the first lanthanum-based endohedral metallofullerene in 1985 with nuclear-shell geometries provided a good environment to further study the interaction between metal and carbon atoms, because the spherical electronic structure of fullerene is resistant to external interaction [14]. Since then, various interatomic interactions have been studied, including metal–metal and metal–nonmetal interactions, in which the types of atoms cover a wide range of atoms, including carbon, oxygen, nitrogen, main group metals, transition metals, lanthanide metal (La-Lu), and actinium metal (Ac-Lr) [15,16,17,18,19]. Particularly, the actinium–carbon interaction is focused on according to the successful isolation of actinium-based metallofullerenes, including U@C_2*n*_ (76 ≤ 2*n* ≤ 86) [20,21], UN@C_82_ [22], Th@C_2*n*_ (76 ≤ 2*n* ≤ 86) [23,24,25], ThC_2_@C_82_ [25], U_2_@C_80_ [26], and Th_2_@C_80_ [27]. However, there has been limited research on the boron–actinium interaction.

Boron chemistry has rapidly developed in the last few decades, including research on elemental boron and boron-based organometallic structure [28,29,30,31,32]. Thus, detailed studies on metal–boron interactions are desired. It is known to all that boron atoms possess similar electronic structures to neighboring carbon atoms, and boron-based nanomaterials have also been developed with similar structures to carbon nanomaterials [33,34,35,36]. A previous study was successfully conducted on the borophene [37], which is a 2D-graphene like structure. An all-boron cage-shaped fullerene nanostructure was also recognized, with similar geometry to fullerenes, in 2014 by Wang and Li [38,39]. This study provided an opportunity to study the nonmetal–boron and metal–boron interactions based on metalloborospherenes. For example, Li et al. theoretically studied the Be-B and Zn-B interactions based on their core-shell octahedral structure [40], and the interaction between boron atoms and transition metal atoms (Sc, Ti, and V atoms) based on transition metal-centered endohedral seashell-like metalloborospherenes [41]. Clearly, previous reports have successfully conducted theoretical studies on the interaction between boron and main group atoms, as well as transition metal atoms [41]. However, there is scarce research on actinide–boron interactions to date. 

In this study, we aimed to investigate the interaction between thorium and boron atoms based on metalloborospherenes by using density functional theory calculations (DFT). Electronic structures are studied by using natural bond orbital (NBO) calculations and frontier molecular orbital analysis. Additionally, metal–nonmetal interactions are studied through bond critical point (BCP) analysis and bond order calculations [42]. The corresponding IR spectrum is simulated in theory to provide useful information for geometrical recognition in future experiments. This research will contribute to a deeper understanding of metal–nonmetal interactions and may lead to the development of new organometallic compounds.

## 2. Results and Discussion

### 2.1. Stability of ThB_40_

**Figure 1:**
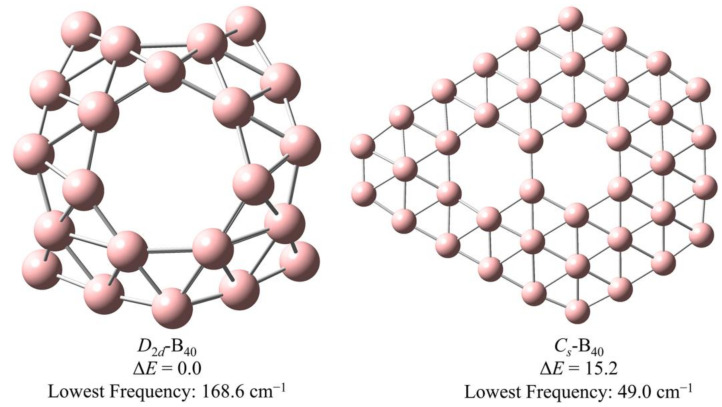
Geometries of *D*_2*d*_-B_40_ and *C_s_*-B_40_ optimized on the TPSSh/6-31G* theoretical level, including their relative energy in kcal/mol and lowest frequency.

The B_40_ isomers, *D*_2*d*_-B_40_ and *C_s_*-B_40_, identified as potential candidates for ThB_40_ compounds following screening with the AM1 semiempirical molecular orbital method [43], were further optimized on the TPSSh/6-31G* theoretical level [44,45]. As shown in Figure 1, the relative energy of *D*_2*d*_-B_40_ was found to be 15.2 kcal/mol lower than that of *C_s_*-B_40_, indicating the enhanced stability of the spherical-like *D*_2*d*_-B_40_ compared to *C_s_*-B_40_. The lowest frequencies recorded were 168.6 cm^−1^ and 49.0 cm-1 for *D*_2*d*_-B_40_ and *C_s_*-B_40_, respectively, indicating the structural stability of both isomers on the potential energy surface, with the potential well of the spherical borospherene *D*_2*d*_-B_40_ being notably deeper than that of the bowl-like *C_s_*-B_40_. The lowest frequency of *D*_2*d*_-B_40_ and *C_s_*-B_40_ also indicates the greater stability of the former isomer. Previous reports on the stability of these two B_40_ isomers align with the outcomes of the present theoretical calculations [39,46]. Furthermore, the electronic spin states of the two stable B_40_ isomers have been considered, confirming the singlet ground state (Table S1), as defined by the energy difference between singlet and triplet states. The energy of *D*_2*d*_-B_40_ and *C_s_*-B_40_ in singlet state is 40.8 kcal/mol and 7.8 kcal/mol lower than that in triplet state, respectively, indicating the singlet-ground electron spin state. The lower energy difference for *C_s_*-B_40_ is attributed to the likely plane geometry, ensuring the share of electrons.

The selected candidate isomers were utilized to form ThB_40_ compounds, and a total of twelve ThB_40_ isomers were considered, as shown in Figure S1. The relative energy for all of these ThB_40_ isomers is shown in Table 1. The findings show that encaged Th into *D*_2*d*_-B_40_ results in the same geometries with the lowest relative energy. Notably, the bowl-like Th@*C_s_*-B_40_ possesses a higher relative energy of 64.2 kcal/mol, indicating the thermodynamic stability of Th-based metalloborospherenes. In order to further confirm the theoretical level, we conducted the optimization of Th@*C_s_*-B_40_ and Th@*D*_2*d*_-B_40,_ as shown in Table S2, and all of the results indicate that Th@*D*_2*d*_-B_40_ is more stable than Th@*C_s_*-B_40_ based on the relative energy, which is in line with the results on the TPSSh/6-31G*~SDD theoretical level. The stable isomer, with the lowest relative energy, possesses an energy gap between the highest occupied molecular orbital (HOMO) and the lowest unoccupied molecular orbital (LUMO) of 0.80 eV, indicating its kinetic stability in a room-temperature environment. 

In order to further confirm the stability of ThB_40_ isomers, the Boltzmann distribution of *three*2-Th@*D*_2*d*_-B_40_ and *hexa*1-Th@*C_s_*-B_40_ was carried out at 1000 K. The results indicate that the concentration of *three*2-Th@*D*_2*d*_-B_40_ exceeds 90%. All of these results strongly support the suitability of *D*_2*d*_-B_40_ as a host for encapsulating the Th atom within its hollow cavity. The following discussion is solely based on the Th-based endohedral metallic borospherene denoted as Th@*D*_2*d*_-B_40_, which serves as an ideal model to further study the interaction between Th and B atoms.

### 2.2. Geometries and Electronic Structures

**Figure 2:**
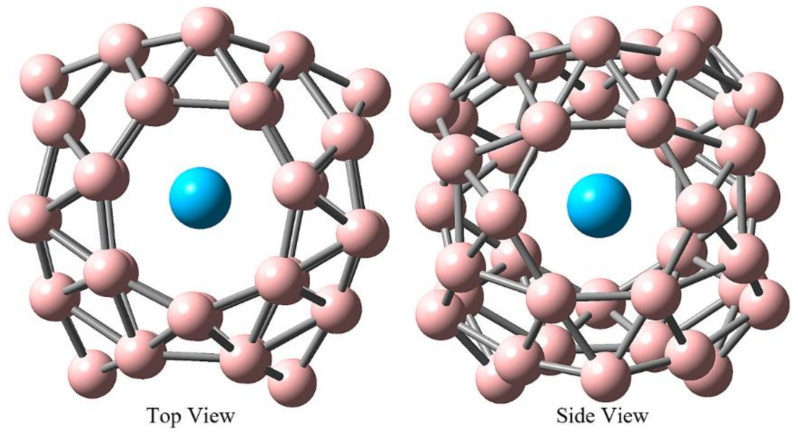
Geometries of optimized Th@*D*_2*d*_-B_40_ on the TPSSh/6-31G*~SDD theoretical level, including top view and side view. The Th and B atoms are colored in blue and pink, respectively.

The nature of molecules is determined by their geometry, and the geometry of stable Th@*D*_2*d*_-B_40_ is shown in Figure 2. The Th atom is situated just off the inner center of the borospherene *D*_2*d*_-B_40_ and lies within a plane of *C_s_* symmetry. The distance between Th and B atoms ranges from 2.94 Å to 3.50 Å, which is larger than the Th-C distances ranging from 2.8 Å to 2.5 Å in Th@C_76_, Th@C_80_, Th@C_82_, and Th@C_86_ [23,27,47,48]. This phenomenon is attributed to the strong coordination of the Th atom with either a pentagon or hexagon in Th-based endohedral metallofullerenes, despite the larger carbon cage. This suggests the weaker interaction of Th-B in endohedral metalloborospherenes compared to Th-C in endohedral metallofullerenes, a point that will be further explored in the following discussions.

The singlet and triplet Th@*D*_2*d*_-B_40_ structures were optimized to determine their spin ground state. The energy of the triplet Th@*D*_2*d*_-B_40_ is 1.5 kcal/mol higher than that of the singlet structure on the TPSSh/6-31G*~SDD level. To further confirm the spin ground state, the energy difference between singlet and triplet states was calculated on the B3LYP, BP86, PBE0, and HSE06 functionals with 6-31G*~SDD, and all of the results in Table S3 indicate the lower relative energy of the singlet state. Under the consideration of the valence electronic structure of the Th atom (6d^2^7s^2^), we inferred a four-electron transfer from Th atom to *D*_2*d*_-B_40_. NBO calculations (shown in Table 2) were carried out to explore the electron transfer and clarify the electronic structures of Th@*D*_2*d*_-B_40_. It is evident that the electrons of 6d and 7s orbitals of the Th atom have been lost, and a four-electron transfer is accepted by the borospherene *D*_2*d*_-B_40_. Consequently, the electronic structure of the endohedral metallic borospherene can be described as Th^4+^@[*D*_2*d*_-B_40_]^4−^. 

**Figure 3:**
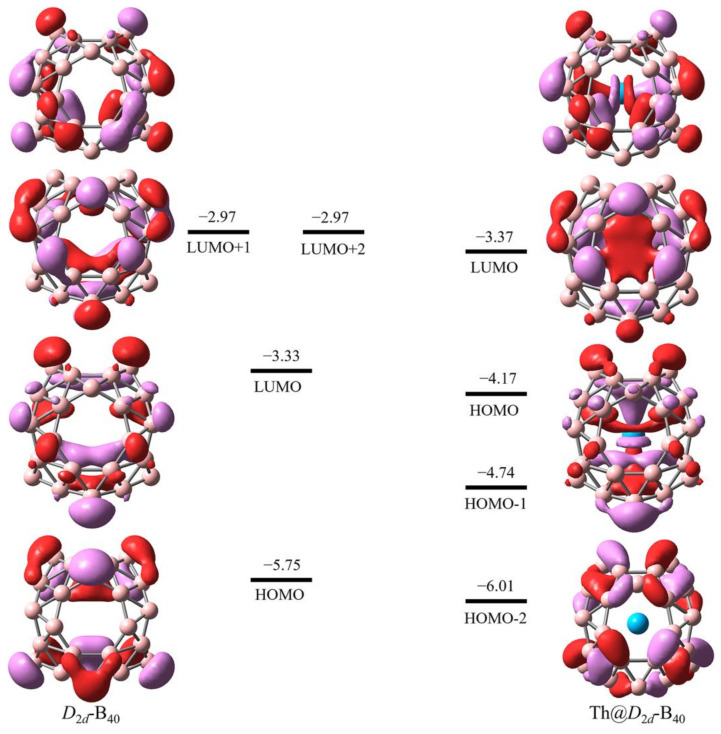
Frontier molecular orbitals of optimized *D*_2*d*_-B_40_ and Th@*D*_2*d*_-B_40_, including the energy level in eV.

As shown in Figure 3, the frontier molecular orbitals have been mapped to further confirm the anticipated four-electron transfer from the Th atom to the B_40_ cage. Notably, two orbitals, LUMO + 1 and LUMO + 2, exhibit degeneracy. The LUMO + 1 and LUMO of the optimized *D*_2*d*_-B_40_ transition to become the HOMO and HOMO-1 of the optimized Th@*D*_2*d*_-B_40_, also providing further evidence of the anticipated four-electron transfer from the Th atom to the borospherene *D*_2*d*_-B_40_ cage. Additionally, orbital overlap is observed in the HOMO and HOMO-1, indicating electron backdonation from the outer cage to the inner metal atom and suggesting a covalent interaction between Th and B atoms. This suggests that electron transition and chemical functionalization will occur in the outer borospherene cage.

### 2.3. Interactions between Th and B_40_

Due to the four-electron transfer, there is a clear electrostatic interaction between Th^4+^ and B_40_^4−^. On the other hand, according to the NBO analysis and the mapping of frontier molecular orbitals, there is a clear electron backdonation from borospherene *D*_2*d*_-B_40_ to the Th atom, suggesting the presence of covalent interaction characteristics. Additionally, Mayer bond orders have been calculated to characterize the covalent interactions between them, as shown in Table 3 [49]. The Mayer bond order ranges from about 0.27 to about 0.34 between the Th atom and B atoms in the outer borospherene *D*_2*d*_-B_40_ cage, with the values of Mayer bond order positively correlating to the distance between Th and B atoms. These calculations indicate the presence of covalent characteristics between the inner metal atom Th and the outer borospherene *D*_2*d*_-B_40_ cage.

**Figure 4:**
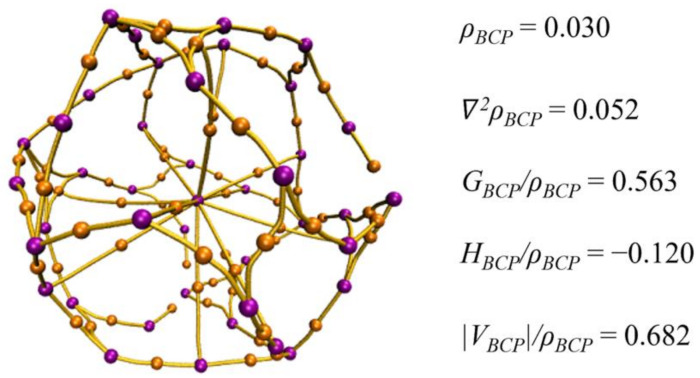
Searching bond critical points of optimized Th@*D*_2*d*_-B_40_, including the average parameters for bond critical points between inner metal atom Th and outer borospherene *D*_2*d*_-B_40_ cage.

To further study the interaction between Th and B_40_, BCP analysis (Figure 4) was carried out [50,51], focusing specifically on the BCPs identified between the Th and B_40_ cages. The detailed parameters of these BCPs are shown in Table S4. In Figure 4, there is the presence of BCPs between Th and the outer borospherene *D*_2*d*_-B_40_ cage, with the average parameters of these BCPs calculated. Generally, the closer the |*V_BCP_*|/*ρ_BCP_* ratio is to 1, the greater the covalent characteristics present, with *V*_BCP_ and *ρ_BCP_* denoting the potential energy density and electronic density of the BCP, respectively. The *ρ_BCP_* value is larger than 0. Additionally, the average value of *H_BCP_*/*ρ_BCP_* is negative, in which *H*_BCP_ is the energy density of the BCP. Accordingly, there is a combination of electrostatic interactions and covalent interactions. In detail, the electrostatic interaction arises from the electron transfer between the inner metal atoms and the outer borospherene cage, while the covalent interaction is attributed to the backdonation from the outer borospherene cage to the inner Th atom. This suggests that the intra-interaction in Th-based endohedral metallic borospherene resembles the ionic model described in endohedral metallofullerens. Clearly, in Th-based endohedral metallic borospherene compounds, the bonding form differs from traditional metal-based coordinated compounds, in which the coordination interaction stems from the electron transfer from organic ligands to the central metal atom, and the electron backdonation occurs from the central metal atom to the organic ligand.

### 2.4. Simulated IR Spectra of D_2d_-B_40_ and Th@D_2d_-B_40_

**Figure 5:**
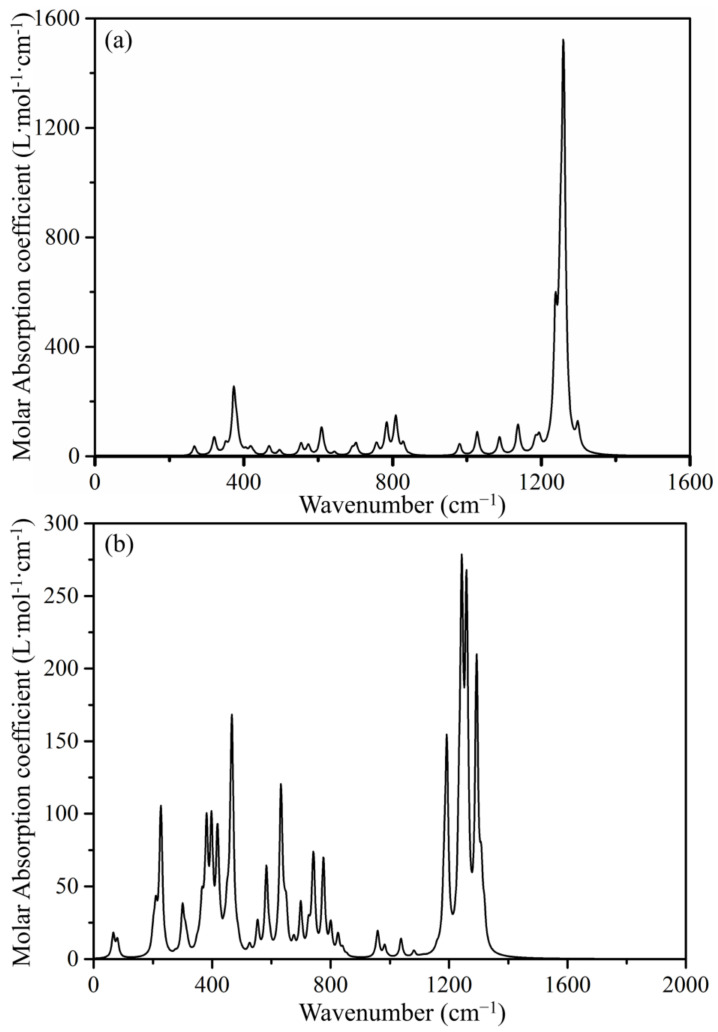
Simulated IR spectra of (**a**) *D*_2*d*_-B_40_ and (**b**) Th@*D*_2*d*_-B_40_ at the TPSSh/6-31G* theoretical level. The broadening function is selected as Lorentzian function, and the width is set as 12 cm^−1^ at half maximum.

Furthermore, the IR spectrum was simulated to provide more information to be able to identify Th@B_40_ in future experiments. The theoretically simulated IR spectra of *D*_2*d*_-B_40_ and Th@*D*_2*d*_-B_40_, as shown in Figure 5 at the TPSSh/6-31G* theoretical level, reveal several significant absorption peaks. Clearly, there are two absorption peaks at round 66 and 79 cm^−1^ for Th@*D*_2*d*_-B_40_ compared with *D*_2*d*_-B_40_. These two absorption peaks correspond to the vibrations which involve the metal atom Th. The maps of displacement vectors, as shown in Figure S3, provide further insight into these vibrations. Around 200 cm^−1^, two absorption peaks are observed, corresponding to the distortion vibration (at about ~187 cm^−1^) and telescopic vibration (at about ~202 cm^−1^). Additionally, clear B-B bonding stretch vibrations are evident at about 1200~1400 cm^−1^, while the absorption peaks in the range of about 400~800 cm^−1^ indicate shearing vibrations of the outer cages. Clearly, the presence of the Th atom not only leads to new featured absorption peaks, but also plays important roles in the vibrations of *D*_2*d*_-B_40_ because of the intra-molecular interaction. These featured absorption peaks in the simulated IR spectrum hold valuable implications for future experiments involving Th-based endohedral metallic borospherenes, offering crucial insights into their structural and vibration properties.

## 3. Calculation Methods

All of the density functional theory calculations in this work were performed by using Gaussian 16 software [52]. The semiempirical molecular orbital (AM1) method was used to preliminarily screen the stability of borospherene B_40_, and several isomers were selected with relative low energy. These selected isomers were further optimized on the TPSSh/6-31G* theoretical level [44,45,53]. The frequency calculations were also carried out on the same theoretical level, free from imaginary frequency for the optimized geometries, indicating the local energy minimum point on the potential energy surface for each optimized isomer. Then, geometry optimizations were carried out for the ThB_40_ isomers on the theoretical levels of TPSSh/3-21G~SDD and TPSSh/6-31G*~SDD [45,54]. Frequency calculations of optimized ThB_40_ were conducted on the TPSSh/6-31G*~SDD theoretical level, also free from imaginary frequency, indicating the potential stability of ThB_40_. In order to confirm the spin ground state of the most stable Th@B_40_, we considered the single and triplet states at the TPSSh/6-31G*~SDD theoretical level. The Boltzmann distribution was carried out at 1000 K according to the equation $\frac{e^{-\Delta E_{i}/RT}}{\sum_{j}e^{-\Delta E_{j}/RT}}$, in which Δ*E* is the relative energy and *T* is the absolute temperature. Frontier molecular orbital and NBO calculations were carried out to analyze the electronic structures [55], and BCP analysis and Mayer bond order (MBO) calculations were used to analyze the bonding characteristics and the interaction characteristics between actinide Th and B_40_ of the most stable Th@B_40_ [49]. The BCP analysis and Mayer bond order calculations are based on the Multiwfn 3.8 software [50]. 

## 4. Conclusions

In conclusion, density functional theory results have revealed that borospherene can serve as a stable carrier for the actinide Th, as demonstrated by Th@B_40_, in which an ionic interaction is uncovered in combination with covalent characteristics between Th and B atoms. The most stable isomer, Th@*D*_2*d*_-B_40_, with a singlet ground state, has been identified. The HOMO-LUMO gap (0.804 eV), defined as the energy difference between the energy of the HOMO and LUMO, and the energy levels of HOMO (−4.18 eV) and LUMO (−3.37 eV) suggest that redox processes can easily occur on the outer borospherene cage. Natural bond orbital analysis has revealed that there is a formal four-electron transfer from the inner Th atom to the outer borospherene B_40_ cage, with backdonation occurring from the outer borospherene cage to the inner metal atom Th. Furthermore, according to the frontier molecular orbital analysis of the hollow borospherene cage and Th-based endohedral metallic borospherene, the electronic configuration has been confirmed as Th^4+^@[*D*_2*d*_-B_40_]^4−^. The NBO and frontier molecular orbital analysis have indicated a significant electrostatic interaction between the inner metal Th atom and the outer borospherene cage. Additionally, the characters of covalent interaction have also been confirmed by the calculation of the MBO between the Th atom and B atom, with the MBO being determined by their distances. The BCP analysis also suggests ionic interaction between the inner metal atom Th and the outer borospherene cage, in combination with covalent characteristics. The simulated IR spectrum has provided valuable information for geometrical identification in future experiments of Th-based endohedral metallic borospherenes. This is expected to enhance our understanding of the interaction between nonmetal atoms and metal atoms, particularly regarding the interaction between B atom and f-metal atoms, and to provide a certain type of guiding information for the research and application of borospherenes.

## Figures and Tables

**Table 1:** Relative energies Δ*E* of ThB_40_ isomers optimized on the TPSSh/6-31G*~SDD theoretical level and their HOMO-LUMO gaps.

Isomers	ΔE (kcal/mol)	Gap (eV)	Isomers	ΔE (kcal/mol)	Gap (eV)
*three*2-Th@*D*_2*d*_-B_40_	0.0	0.80	*hexa*1-Th@*C_s_*-B_40_	64.2	0.86
*hepta*-Th@*D*_2*d*_-B_40_	0.0	0.80	Th@*C_s_*-B_40_	66.6	1.0
*three*-Th@*D*_2*d*_-B_40_	0.0	0.80	*three*-Th@*C_s_*-B_40_	80.9	0.87
*two*-Th@*D*_2*d*_-B_40_	0.0	0.80	*hexa*2-Th@*C_s_*-B_40_	91.7	0.97
*center*-Th@*D*_2*d*_-B_40_	0.0	0.80	-	-	-
*hexa*-Th@*D*_2*d*_-B_40_	0.0	0.80	-	-	-
*hexa*2-Th@*D*_2*d*_-B_40_	84.2	0.98	-	-	-
*hexa*3-Th@*D*_2*d*_-B_40_	91.6	1.12	-	-	-

**Table 2:** Natural population analysis for optimized Th@*D*_2*d*_-B_40_, including Th and B atoms in which the targeted B atom is closest to Th atom, on the TPSSh/6-31G*~SDD theoretical level.

Atoms	Populations
Th	7s^0.01^5f^0.37^6d^0.14^7p^0.52^8s^0.19^
B	2s^0.53^2p^2.45^3s^0.01^3p^0.02^

**Table 3:** Mayer bond order between Th atom and several B atoms close to the metal atom (Figure S2), and their bond length in Å in optimized Th@*D*_2*d*_-B_40_ on the TPSSh/6-31G*~SDD theoretical level.

Bonds	Mayer Bond Order	Bond Length
Th-B7	0.342	2.94
Th-B10	0.269	3.26
Th-B17	0.342	2.94
Th-B32	0.290	2.97

## Data Availability

The data that support the findings of this study are available from the corresponding authors upon reasonable request.

## Supplementary Materials

The following supporting information can be downloaded at: https://www.mdpi.com/article/10.3390/molecules29061222/s1, Figure S1: Geometries of potential isomers of ThB_40_ based on *D*_2*d*_-B_40_ and *C_s_*-B_40_ isomers, Table S1: relative energy of *D*_2*d*_-B_40_ and *C_s_*-B_40_ in different spin multiplicities, Tables S2 and S3: relative energy of Th@*D*_2*d*_-B_40_ and Th@*C_s_*-B_40_ at different functionals, and relative energy of Th@*D*_2*d*_-B_40_ in different spin multiplicities. Figures S2 and S3, Table S4: Geometry of Th@*D*_2*d*_-B_40_ with atom labels, parameters of bond critical points, featured displacement vectors of Th@*D*_2*d*_-B_40_, and coordinates of optimized geometries. Coordinates of *D*_2*d*_-B_40_, *C_s_*-B_40_, Th@*D*_2*d*_-B_40_, and Th*C_s_*-B_40._
