# Supplementary material for: Insights into ThB40: Stability, Electronic Structure, and Interaction
Source: Molecules. 2024 Mar 8;29(6):1222. doi: 10.3390/molecules29061222 (PMC10975232; doi:10.3390/molecules29061222)
Supplement: Supplementary file 1 [file molecules-29-01222-s001.zip › molecules-2882488-supplementary.pdf]

Supporting Information

Insights into ThB_{40}: Stability, Electronic Structure, and Interaction

Yutian Li [1,*], Yingying Wang [1], Zhanrong Zhou [1], Yang Gao [1], Yiming Chen [1], Guoqing Zhang [1] and Chao Ma [2]

[1] Xi'an Research Institute of High Technology, Xi'an 710025, China; gaoyang_nudt@126.com (Y.G.)
[2] Foundation Department, Engineering University of PAP, Xi'an 710086, China
* Correspondence: lyt13593587864@stu.xjtu.edu.cn

Contents:

Figure S1 Potential isomers of ThB_{40} based on D_{2d}-B_{40} and C_s-B_{40} isomers………...S3

Table S1 Relative energy of D_{2d}-B_{40} and C_s-B_{40} in different spin multiplicities…….S4

Table S2 Relative energy of Th@D_{2d}-B_{40} and Th@C_s-B_{40} at different functionals.....S4

Table S3 Relative energy of Th@D_{2d}-B_{40} in different spin multiplicities..................S4

Figure S2 Geometry of Th@D_{2d}-B_{40} with atom labels...S5

Table S4 Parameters of bond critical points between B and Th atoms………………S5

Figure S3 Maps of feature displacement vectors in Th@D_{2d}-B_{40}...............................S6

Coordinates of D_{2d}-B_{40}, C_s-B_{40}, Th@D_{2d}-B_{40}, and ThC_s-B_{40}......................................S7

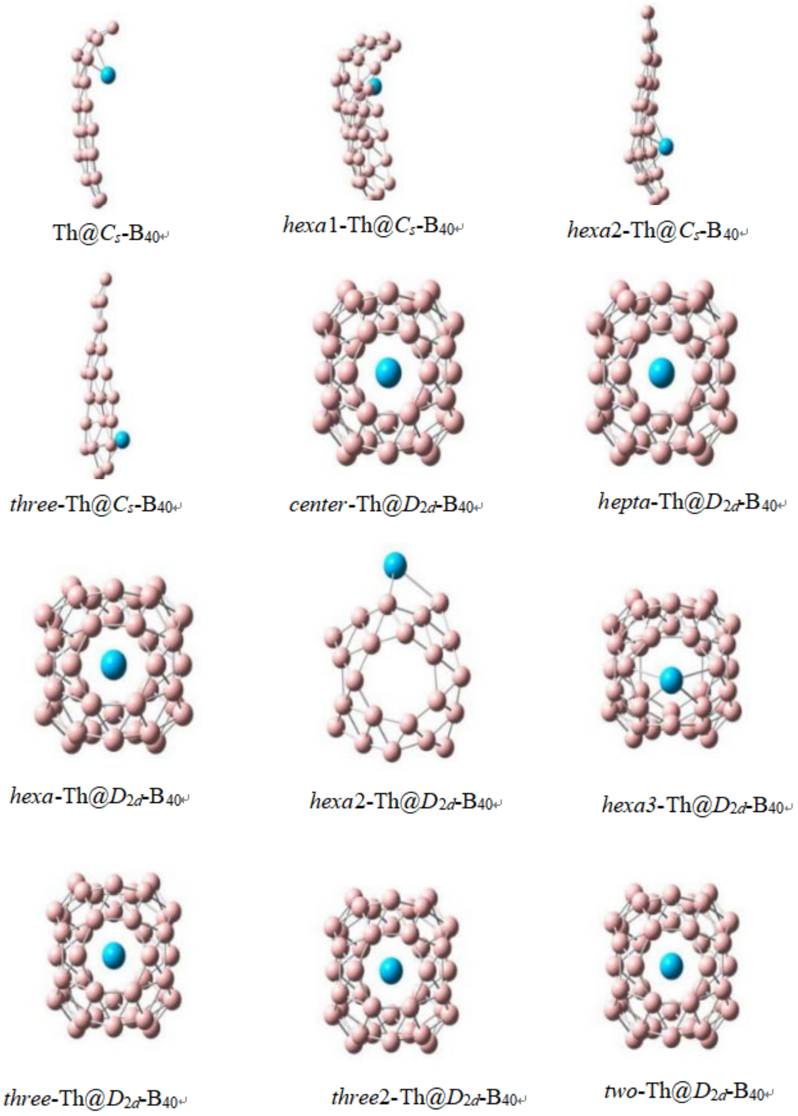

Figure S1 Potential isomers of ThB_{40} based on D_{2d}-B_{40} and C_s-B_{40} isomers optimized on the TPSSh/6-31G*~SDD. The Th atoms are colored in blue and B atoms are colored in pink.

Table S1 Relative energy of D_{2d}-B_{40} and C_s-B_{40} in different spin multiplicities optimized on the the TPSSh/6-31G* theoretical level.

Isomers	Singlet/a.u.	Triplet/a.u.	ΔE/kcal·mol^{-1}
D_{2d}-B_{40}	-994.035351	-993.970392	40.8
C_s-B_{40}	-994.011059	-993.998578	7.8

Table S2 Relative energy (kcal·mol^{-1}) of Th@D_{2d}-B_{40} and Th@C_s-B_{40} at different functionals with 6-31G*~SDD basis set.

Isomers	B3LYP	BP86	PBE0	HSE06
Th@D_{2d}-B_{40}	0.0	0.0	0.0	0.0
Th@C_s-B_{40}	29.2	60.5	65.0	61.3

Table S3 Relative energy (kcal·mol^{-1}) of Th@D_{2d}-B_{40} in different spin multiplicities optimized on the different functionals with 6-31G*~SDD basis set.

Isomers	B3LYP	BP86	PBE0	HSE06
Singlet	0.0	0.0	0.0	0.0
Triplet	1.7	1.8	1.0	1.0

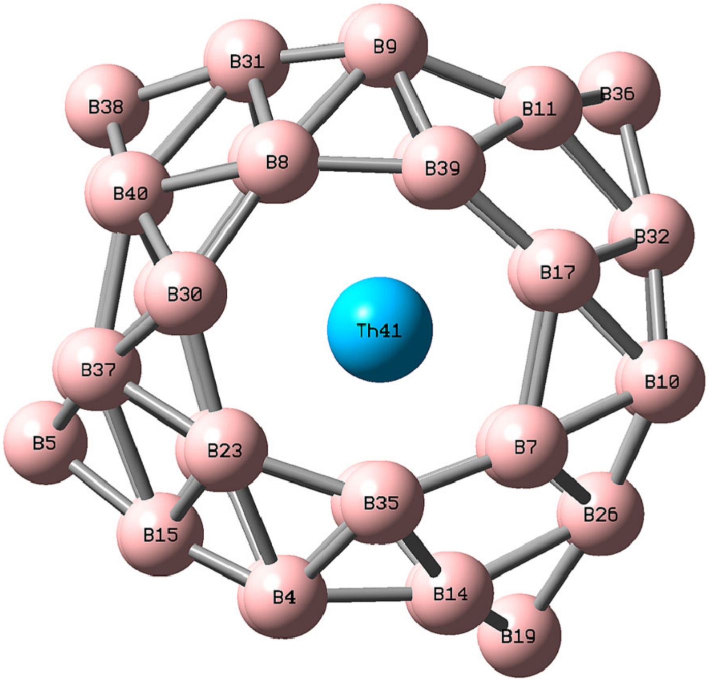

Figure S2 Geometry of Th@D_{2d}-B_{40} with atom labels.

Table S4 Parameters of bond critical points between B and Th atoms.

	P_{BCP}	$\nabla^2\rho_{BCP}$	G_{BCP}/ρ_{BCP}	H_{BCP}/ρ_{BCP}	$\lvert V_{BCP}\rvert/\rho_{BCP}$
Th-B7	0.033	0.055	0.566	-0.147	0.713
Th-B8	0.025	0.052	0.602	-0.090	0.692
Th-B15	0.026	0.044	0.520	-0.094	0.614
Th-B17	0.033	0.055	0.566	-0.147	0.713
Th-B32	0.031	0.054	0.560	-0.120	0.680

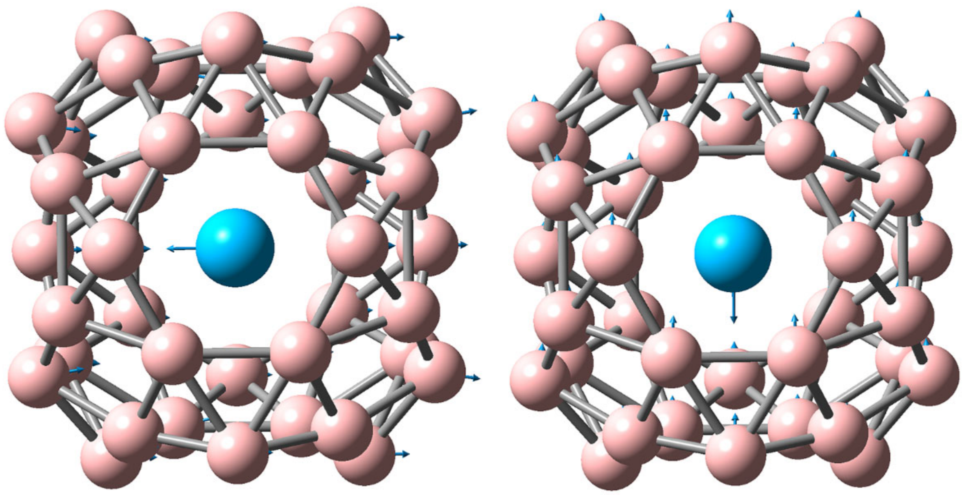

Figure S3 Maps of feature displacement vectors in Th@D_{2d}-B_{40} simulated on the TPSSh/6-31G*~SDD. The Th atoms are colored in blue and B atoms are colored in pink. The vectors are marked by blue arrows.

Coordinates of D_{2d}-B_{40}, C_s-B_{40}, Th@D_{2d}-B_{40}, and ThC_s-B_{40}

D_{2d}-B_{40}

B	1.75260100	2.74276900	-0.38394100
B	1.69437100	0.00000000	2.87638700
B	1.39005000	2.37032000	1.20490500
B	-1.75260500	-2.74277100	-0.38394100
B	0.00000100	-1.69437400	-2.87638600
B	2.65161400	0.00000100	-1.99487700
B	-2.37185900	-0.87949400	1.68046200
B	-2.37031700	1.39004900	-1.20490700
B	-1.75260400	2.74277100	-0.38394500
B	-1.69437000	0.00000100	2.87638600
B	-1.39004900	2.37031900	1.20490100
B	2.74277000	-1.75259000	0.38394200
B	1.39005400	-2.37031900	1.20490800
B	-1.39005100	-2.37031900	1.20490400
B	-0.87948800	-2.37184400	-1.68044200
B	0.87948800	2.37184400	-1.68044900
B	-2.37185600	0.87949500	1.68045800
B	0.87949800	-2.37185700	-1.68046100
B	0.00000000	-2.65161400	1.99486400
B	2.74277000	1.75259100	0.38394300
B	2.37185800	0.87949500	1.68046200
B	2.37185100	-0.87949600	1.68045900
B	-2.37032000	-1.39004900	-1.20490400
B	2.37031500	1.39004900	-1.20490400
B	2.37032400	-1.39004800	-1.20490700
B	-0.88977100	-1.39150500	2.54058300
B	0.88976900	-1.39150600	2.54058900
B	1.75259700	-2.74276600	-0.38394200
B	1.39150100	-0.88976700	-2.54058100
B	-2.65161100	0.00000000	-1.99487600
B	-0.87949000	2.37184500	-1.68045300
B	-0.88977000	1.39150600	2.54058200

C_s-B_{40}

B	-0.38248100	2.17586700	1.71409600
B	0.09764200	2.15632900	3.36305000
B	0.23227700	0.73390000	4.19430100
B	-0.23130500	-0.71027900	3.33041300
B	-0.69718300	-0.70405000	1.65693800
B	-0.75186600	0.74848000	0.82571100

B	-0.67252300	-2.16161100	0.82965500
B	0.36686500	-2.11286500	4.12071900
B	0.19624400	3.46064000	0.84034600
B	-0.29084600	-2.12673000	2.47346200
B	0.22324500	3.54168300	2.54492300
B	0.50418700	-0.68845900	4.81932600
B	0.41668700	-3.55737000	3.28810400
B	-0.13570900	-3.57567800	1.65830800
B	0.42471500	-4.98658300	0.82813100
B	-0.69717300	-0.70404800	-1.65693600
B	-0.29084100	-2.12673000	-2.47345600
B	-0.13578300	-3.57569400	-1.65831400
B	0.42470900	-4.98657900	-0.82814100
B	0.65049000	-4.84008600	-2.39369700
B	0.41651300	-3.55742700	-3.28814800
B	0.36698800	-2.11285500	-4.12066700
B	-0.23126800	-0.71028100	-3.33040500
B	0.50430500	-0.68845200	-4.81927900
B	0.09763800	2.15632800	-3.36305000
B	0.23224800	0.73390800	-4.19432000
B	-0.38247000	2.17586300	-1.71409300
B	0.22327200	3.54167600	-2.54491700
B	0.19625000	3.46063900	-0.84034300
B	-0.47971100	0.76066200	-2.49335000
B	-0.47971200	0.76066300	2.49334800
B	-0.30456500	-3.59595800	0.00000100
B	-0.75187000	0.74847900	-0.82571200
B	0.65068900	-4.84002100	2.39365100
B	0.26565800	4.96766000	1.71314800
B	-0.09146000	4.89717300	-0.00000100
B	0.26567300	4.96765700	-1.71314800
B	0.46150700	6.26788100	-0.78390100
B	0.46150500	6.26788100	0.78390000
B	-0.67253700	-2.16161300	-0.82965400

Th@D_{2d}-B_{40}

B	1.66177800	2.77627300	-0.36578500
B	1.60870000	0.00074000	2.93216200
B	1.36858700	2.44459100	1.25574500
B	-1.70480900	-2.77450100	-0.36486800
B	-0.02229600	-1.69473500	-2.94504400
B	2.53853100	0.00048700	-1.89295200
B	-2.32762700	-0.88028600	1.70746400

B	-2.34809700	1.45357400	-1.21866200
B	-1.70464800	2.77612700	-0.36518000
B	-1.65362700	0.00093500	2.93325400
B	-1.41344200	2.44513100	1.25691500
B	2.61450500	-1.68626900	0.39450300
B	1.36836900	-2.44327700	1.25601500
B	-1.41389000	-2.44331700	1.25726900
B	-0.87062300	-2.38460200	-1.66639800
B	0.82696500	2.38565100	-1.66717000
B	-2.32761600	0.88183700	1.70725800
B	0.82674900	-2.38451300	-1.66693900
B	-0.02241100	-2.73137900	2.01704600
B	2.61461700	1.68737900	0.39427400
B	2.28390000	0.88166100	1.70696300
B	2.28395600	-0.88041700	1.70717000
B	-2.34830700	-1.45209500	-1.21857600
B	2.30436400	1.45348800	-1.21898600
B	2.30431800	-1.45240200	-1.21880000
B	-0.87584600	-1.39955900	2.58826500
B	0.83207100	-1.40068300	2.58793100
B	1.66151000	-2.77501800	-0.36551200
B	1.33017300	-0.88573900	-2.57784600
B	-2.58256300	0.00073200	-1.89309300
B	-0.87039000	2.38592100	-1.66662200
B	-0.87571700	1.40126100	2.58793800
B	0.83225100	1.40224900	2.58785800
B	1.33020800	0.88670900	-2.57791500
B	-2.65854800	-1.68591700	0.39463300
B	-0.02210100	2.73297800	2.01693200
B	-1.37481100	-0.88598700	-2.57796700
B	-0.02214600	1.69587700	-2.94521400
B	-2.65831800	1.68764400	0.39451400
B	-1.37471300	0.88724400	-2.57805200
Th	-0.02260900	0.00062100	0.11119300

ThC_s-B_{40}

B	-0.33628900	1.90723400	1.62408600
B	0.41432400	1.71381300	3.20409900
B	0.11321800	0.43842300	4.17044400
B	-0.57326900	-0.92232900	3.32651700
B	-1.03296100	-0.86951500	1.67471600
B	-1.09236600	0.59868700	0.85839700
B	-0.94851500	-2.27884600	0.80754700

B	-0.23068800	-2.38789300	4.16082100
B	-0.26419100	3.46578600	0.96415400
B	-0.84756900	-2.34710400	2.50525600
B	0.86640400	2.95686700	2.28118800
B	0.08835900	-0.98014200	4.86169500
B	-0.08803900	-3.76445500	3.29375900
B	-0.38289300	-3.68232800	1.57662000
B	0.56248100	-4.85929400	0.76493200
B	-1.13940100	-0.60270300	-1.57730400
B	0.37872500	-1.41082700	-1.93871100
B	0.44108600	-3.08528500	-1.55196100
B	1.11014700	-4.46101600	-0.74076100
B	1.87043500	-3.84426200	-1.96264800
B	1.63423500	-2.44915000	-2.67228100
B	1.37349600	-0.95924900	-3.26476600
B	-0.21103200	-0.20859600	-2.98534900
B	1.04015300	0.45662000	-3.90519900
B	-1.27378800	2.38971100	-3.04070400
B	-0.17541300	1.42536400	-3.69626000
B	-1.80177700	2.26562700	-1.40221900
B	-1.33600400	3.70598100	-2.12042600
B	-1.01145000	3.73789500	-0.44772200
B	-1.44470700	0.86120700	-2.34460600
B	-0.66873100	0.56399800	2.52219800
B	-0.25217600	-3.52898600	-0.07908800
B	-1.70300100	0.79196900	-0.65795100
B	0.44397200	-4.91057400	2.34506500
B	1.59947200	3.78431400	1.13120400
B	0.57271900	4.43734000	-0.15247400
B	-0.05719100	4.58917500	-1.73759500
B	1.51361200	4.74446300	-1.51349000
B	2.26308000	4.28375600	-0.23126100
B	-0.76784300	-2.07319400	-0.84529000
Th	0.81486900	1.87567200	-1.07554100
